# Influence of Habitual Dairy Food Intake on LDL Cholesterol in a Population-Based Cohort

**DOI:** 10.3390/nu13020593

**Published:** 2021-02-11

**Authors:** Silvio Buscemi, Davide Corleo, Carola Buscemi, Cristiana Randazzo, Antonio Maria Borzì, Anna Maria Barile, Giuseppe Rosafio, Marcello Ciaccio, Rosalia Caldarella, Francesco Meli, Salvatore Maestri, Walter Currenti, Raffaele Ivan Cincione, Paolo Murabito, Fabio Galvano

**Affiliations:** 1 Dipartimento di Promozione della Salute, Materno-Infantile, Medicina Interna e Specialistica di Eccellenza (PROMISE), University of Palermo, I-90127 Palermo, Italy; davidecorleo@gmail.com (D.C.); carola.buscemi@gmail.com (C.B.); randazzocristiana@yahoo.it (C.R.); antoniomaria.borzi@unipa.it (A.M.B.); annamaria.barile@unipa.it (A.M.B.); giuseppe.rosafio@unipa.it (G.R.); rosalia.caldarella@policlinico.pa.it (R.C.); francesco.meli@unipa.it (F.M.); salvatore.maestri@unipa.it (S.M.); 2 Unit of Clinical Nutrition, AOU Policlinico “P. Giaccone”, I-90127 Palermo, Italy; 3 Dipartimento di Biomedicina, Neuroscienze e Diagnostica Avanzata (BIND), University of Palermo, I-90127 Palermo, Italy; marcello.ciaccio@unipa.it; 4 Unit of Laboratory Medicine, AOU Policlinico “P. Giaccone”, I-90127 Palermo, Italy; 5 Dipartimento di Scienze Biomediche e Biotecnologiche, University of Catania, I-95100 Catania, Italy; currentiw@gmail.com (W.C.); fgalvano@unict.it (F.G.); 6 Dipartimento di Medicina Clinica e Sperimentale, University of Foggia, I-71100 Foggia, Italy; ivan.cincione@unifg.it; 7 Dipartimento di Chirurgia Generale e Specialità Medico-Chirurgiche, University of Catania, I-95100 Catania, Italy; paolo.murabito@gmail.com

**Keywords:** dairy, milk, ricotta, LDL, cholesterol

## Abstract

Background: Cholesterol has a pivotal role in human physiology, exerting both structural and functional activity. However, higher blood cholesterol levels, especially low-density lipoprotein cholesterol (LDL-C), are a major cardiovascular risk factor. Therefore, special attention has been given to the effect of dietary factors in influencing LDL-C blood levels. In particular, much research has focused on dairy products, since they are a main component of different dietary patterns worldwide. A large body of evidence did not support the hypothesis that dairy products significantly increase circulating LDL-C, but no definitive data are available. Hence, we aimed to assess the relationships among LDL-C, habitual dairy food intake and anthropometric variables in a cohort representative of the general population in a Mediterranean area. Methods: We evaluated 802 healthy adults included in the ABCD_2 (Alimentazione, Benessere Cardiovascolare e Diabete) study (ISRCTN15840340), a longitudinal observational single-center study of a cohort representative of the general population of Palermo, Sicily. The habitual intake of dairy products was assessed with a validated food frequency questionnaire, and LDL-C serum levels and several anthropometric parameters were measured. Results: The group with high LDL-C serum concentrations (≥130 vs. <130 mg/dL) exhibited higher age, body mass index (BMI), waist-to-hip ratio (WHR), body fat percentage, systolic and diastolic blood pressure, carotid intima-media thickness and glycated hemoglobin. The habitual diet was not different between the groups in terms of macronutrient, cholesterol, egg and dairy food intake, with the exception of the weekly number of portions of milk (higher in the low LDL-C group vs. the high LDL-C group) and ricotta cheese (higher in the high LDL-C group vs. the LDL-C group). No significant correlation was found between LDL-C blood levels and the habitual intake of dairy products or the dietary intake of cholesterol and fats. The multivariate regression analyses (R^2^ = 0.94) showed that LDL-C blood levels were significantly associated with the habitual intake of milk (*p* < 0.005) and ricotta cheese (*p* < 0.001) and with BMI (*p* < 0.001). Conclusion: Our study reported that total dairy food consumption was not correlated with LDL-C blood levels. However, multivariate analyses showed an inverse association between serum LDL-C and milk intake as well as a positive association between ricotta cheese intake and LDL-C concentrations. More studies are needed to better characterize the relationship between dairy products and circulating LDL-C.

## 1. Introduction

Cardiovascular diseases (CVDs) are the main cause of death worldwide, representing nearly 25% of all deaths in both developing and developed countries [1]. The economic burden of CVD is enormous, with costs that are estimated to be more than 200 billion dollars per year, both in Europe [2] and in the United States [1]. One of the major modifiable risk factors for CVD is the concentration of blood cholesterol and its lipoprotein subclasses, since the deposition of cholesterol in the arterial walls was demonstrated as the key initiating event of atherosclerosis [3]. In particular, blood levels of low-density lipoprotein cholesterol (LDL-C) are mainly responsible for atherosclerosis promotion and progression [4], and the strong association between serum LDL-C and CVD is well acknowledged [5]. Therefore, targeting low LDL-C serum levels is a cornerstone for cardiovascular prevention and treatment [6]. Cholesterol supply is determined by both dietary intake (exogenous production) and de novo biosynthesis (endogenous production), the former accounting for approximately 30% of total circulating cholesterol [7]. Hence, dietary intake has a pivotal role in influencing serum LDL-C concentrations, and many studies have demonstrated that dietary patterns are associated with blood lipid profiles [8]. In particular, dairy products include foods with high nutritional value that are consumed worldwide as fundamental components of several dietary patterns. Therefore, special attention has been given to the habitual intake of dairy foods and the effects on serum LDL-C concentrations [9,10]. In particular, although the bulk of the studies reported that dairy food intake was not associated with higher LDL-C serum concentrations [11,12,13,14,15,16], no definitive and unequivocal data are available since other studies found a positive [17] or even a negative association [18]. Factors as age [11], gender [12], concomitant CVD, diabetes or use of cholesterol-lowering drugs [14,15,16] might be potential confounding factors in studies exploring the influence of dietary factors on cholesterolemia. Consequently, more studies are required, particularly those investigating large cohorts representative of the general healthy population, to better characterize this relationship, especially considering the consequences for human health and the significant substantial resources invested in facing CVD. Therefore, our aim was to investigate the relationship between LDL-C serum concentrations, anthropometric parameters and the habitual intake of dairy foods in a cohort of randomly selected healthy adults living in the Mediterranean area of Palermo (Sicily, Italy).

## 2. Materials and Methods

### 2.1. Participants

The Nutrition, Cardiovascular Wellness and Diabetes (ABCD_2) project (ISRCTN15840340) is a longitudinal observational single-center study of a cohort representative of the general population living in Palermo, the largest city in Sicily (Italy), with a population of 674,742. The ABCD study cohort was recruited in 2011, as previously described [19]. In particular, the inclusion criteria were participants aged >18 years who resided in Palermo. The demographic characteristics of the ABCD cohort were similar to, if not overlapping with, those of the general population of the same age range (18–90 years) living in Palermo in 2011, as presented elsewhere [20]. The original cohort was recontacted (telephone, e-mail, letter) in 2015, and those who agreed to participate in the study were asked to come to the Metabolism and Clinical Nutrition Laboratory of the Department of Internal and Specialized Medicine at the University of Palermo and were reexamined from 21 March to 31 July. Each participant was given the opportunity to present a relative or friend as a new participant in the study, but the number of new participants was limited to the first 300 people. The demographic characteristics of the ABCD_2 cohort were not significantly different from those of the ABCD_1 cohort, as presented elsewhere [21]. Limited to the present study, people with known diabetes, coronary heart disease, previous stroke and current use of statins or other lipid-lowering drugs were excluded from calculations.

The institutional Ethics Committee (“Palermo 1” of the Policlinico “P. Giaccone” University Hospital, 3 November 2014, ref: March 2015) approved the study protocol, and each participant signed an approved informed consent form.

Participants were administered questionnaires on demographic characteristics, the presence of chronic diseases and pharmacologic treatment. Additionally, a Food Frequency Questionnaire and a questionnaire on habitual physical activity were administered. All participants underwent anthropometric, body composition, and carotid intima-media thickness (c-IMT) measurements, and a postabsorptive fasting blood venous sample was obtained for laboratory tests.

### 2.2. Food Frequency Questionnaire

Half-quantitative habitual intake of different foods during the past 12 months was assessed with a previously validated local population medium-length Food Frequency Questionnaire (FFQ) as reported elsewhere [22]. Specifically, participants were asked by trained dietitians about their consumption of 36 different food items and were also asked to indicate their usual rate of consumption, choosing from seven frequency categories, ranging from “never” or “less than once a week” to “7 times per week.” The food items were categorized as drinks, cow milk and dairy products, meat-fish-eggs, cereals, vegetables-legumes-fruit, fat dressing, and other (sweets, fried foods and fast food). For coffee, alcoholic and soft drinks, the amount in terms of cups/glasses was also specified. In most instances, a reference food was considered for different items, and food photographs were used to help in portion-size description [23]; requested data referred to the last year. Data are expressed as the number of portions consumed (daily or weekly) of each food and as an estimation of diet composition.

Additionally, a specifically developed and validated questionnaire was administered to define the individual level of habitual physical activity (HPA) as described elsewhere [21]. Briefly, on the basis of both the intensity and frequency of physical exercise, 4 different levels of HPA were defined: (a) very low (no habitual exercise); (b) low (walks of 20–30 min at least 3 times a week); (c) medium (scheduled physical activity 1–3 times a week, or walks >30 min at least 3 times a week); and (d) high (scheduled physical activity >3 times a week or competitive sport activity or heavy job activity). For the purposes of the study, sedentary people were defined as those with very low or low HPA, and nonsedentary people were defined as those with medium or high HPA.

### 2.3. Measurements

Height and body weight were measured with participants lightly dressed and without shoes (SECA); the body mass index (BMI) was calculated as body weight (kg)/height^2^ (m^2^). Body circumferences were measured at the umbilicus (waist circumference) and at the most prominent buttock level (hip circumference); the ratio (waist-to-hip ratio [WHR]) was used as an indirect index of body fat distribution [24]. Systolic and diastolic arterial blood pressure (two measurements obtained at 5-minute intervals in a seated position) and heart rate (Omron M6; Omron Healthcare Co; Matsusaka, Mie, Japan) were measured by physicians or dietitians according to standardized procedures. Body composition in terms of fat mass (FM) and fat-free mass (FFM) was estimated using bioelectrical impedance analysis (BIA; BIA-101 Anniversary, Akern; Florence, Italy) following the manufacturer’s equations, as previously described [25].

### 2.4. Laboratory Analysis

Participants underwent blood sampling for the assessment of blood chemistry and hormonal parameters. For each participant, a blood sample was frozen and stored at −80°C for subsequent measurements. Fasting plasma glucose (FPG), total cholesterol, high-density lipoprotein cholesterol (HDL-C), triglycerides (Tg), uric acid and creatinine concentrations were ascertained using standard clinical chemistry methods (Glucosio HK UV; Colesterolo tot. Mod P/D; Colesterolo HDL gen 3 mod P/917; Trigliceridi; Acido urico MOD P/917; Creatinina enzimatica; Roche Diagnostics; Monza, Italy). Basal insulin concentrations (Elecsys insulina; Roche Diagnostics; Monza, Italy), high sensitivity c-reactive protein (hs-CRP; B-analyst hs-CRP; Menarini diagnostics; Florence, Italy) and glycated hemoglobin (HbA1c; B-analyst HbA1c; Menarini diagnostics; Florence, Italy) were also measured. LDL-C serum concentration was calculated with Friedewald’s formula [26], and HOMA-IR was calculated as described by Matthews et al. [27].

### 2.5. Statistical Analysis

Data are reported as the means ± SD for continuous variables and as percentages for categorical variables. Normal distribution of each variable was tested using the Shapiro–Wilk test, variables with skewed distribution were log-transformed when appropriate. LDL-C values were also categorized as “<130” and “≥130” mg/dL according to the values that were historically considered normal for primary prevention of cardiovascular events [28]. Student’s *t*-test for unpaired data was used to compare continuous variables between two groups. The χ^2^ test was used to compare categorical variables. Pearson’s r correlation coefficients were calculated to explore the associations among variables. Factors that were significantly correlated with LDL-C values were included in stepwise multivariate regression (backward model selection) analysis. A two-tailed *p* value < 0.05 was considered significant. All analyses were performed with Systat (Windows version 13.0; San Jose, CA, USA).

## 3. Results

A total of 1033 participants (415 males and 618 females) were initially selected. Among these, 225 participants were excluded because of type 2 diabetes, coronary heart disease, previous stroke or use of statins or other lipid-lowering drugs (including fibrates, ezetimibe, proprotein convertase subtilisin/kexin type 9 inhibitors, and omega-3 fatty acids) in different combinations, four cases were excluded because of incomplete data, and 2 cases were excluded because of pregnancy. Finally, a total of 802 participants were evaluated. The physical, biochemical, and clinical characteristics of the participants divided into two groups according to LDL-C blood concentrations (<130 and ≥130 mg/dL) are presented in Table 1. The group with high LDL-C blood concentrations exhibited higher age, BMI, WHR, body fat, systolic and diastolic blood pressure, c-IMT and glycated hemoglobin. The group with high LDL-C also had a higher prevalence of menopausal women (64.1 vs. 35.9%; *p* < 0.001). Concerning the habitual diet, as summarized in Table 1, no difference was observed between the groups in terms of the intake of macronutrients, cholesterol, eggs and dairy foods with the exception of the weekly number of portions of cow milk and ricotta cheese that were higher in the low LDL-C group and the high LDL-C group, respectively. No difference was observed considering the number of portions consumed of fresh or aged cheese, yogurt, or mozzarella cheese. A similar number of people did not habitually consume milk in the two groups (37.0% vs. 44.9%, low vs. high LDL-C group; *p*= 0.10); furthermore, no difference was observed concerning the use of whole milk (6.1 vs. 5.2%), low-fat milk (53.5 vs. 45.5%) and skimmed milk (3.4 vs. 4.4%). The variables BMI, fat mass, waist and hip circumferences, habitual intake of cholesterol, fats, milk and ricotta cheese were not normally distributed and, consequently, log transformed. Simple correlations between LDL-C blood concentrations and anthropometric and diet variables are presented in Table 2. The multiple regression analysis demonstrated that age, BMI, habitual intake of milk and ricotta cheese (but not daily cholesterol intake, fat mass, waist circumference and WHR) were independently associated with blood LDL-C concentrations (Table 3).

## 4. Discussion

This study found that in a cohort of a general real-life population, habitual dairy food intake and LDL-C blood levels were not associated. However, we observed some exception. In fact, the habitual intake of cow milk was inversely associated with serum LDL-C concentrations, while that of habitual ricotta cheese was positively associated with LDL-C. The lack of correlation between dairy products and LDL-C is not surprising, and other studies reported similar findings. Barr and colleagues showed that increasing milk consumption did not increase LDL-C concentrations [11]. Similarly, Beavers et al. observed that 4 weeks of regular milk consumption did not increase LDL-C compared with soy milk beverages [12]. Furthermore, in a cohort of postmenopausal women, no significant difference in LDL-C blood levels was found between individuals consuming milk and those using a milk-free diet [14]. Again, Wade and colleagues analyzed a cohort of individuals randomized to consume a Mediterranean diet supplemented with dairy products and a low-fat diet, reporting no difference in circulating LDL-C between the two groups [15]. Conversely, another study reported that increasing dairy product consumption slightly, yet significantly, increased LDL-C levels [17]. Intriguingly, a negative association was also found between dairy product consumption and LDL-C serum levels [18]. However, a recent meta-analysis [13] that included nine randomized controlled trials (RCTs) reported that low-fat as well as high-fat dairy food diets did not significantly influence circulating LDL-C. Regarding milk intake, although many studies have investigated the relationship between milk consumption and LDL-C concentrations, there are inconsistencies. Our findings are in agreement with Rossouw and colleagues who reported that abundant skim milk consumption significantly lowered LDL-C in a group of healthy adolescents [29]. Similarly, a study evaluating a cohort of more than 3000 subjects reported that individuals with higher milk consumption had lower LDL-C blood levels [18]. Furthermore, it was demonstrated that increasing milk consumption induced no significant change in blood concentrations of LDL-C [11]. Comparable findings were obtained by Drouin-Chartier et al., who found that a milk-providing diet did not increase LDL-C serum levels compared with a milk-free diet [14]. Similar results were obtained by Bard et al., who performed a RCT and demonstrated no correlation between LDL-C serum levels and milk-containing diets, even in comparison with vegetable fat-containing consumption [16]. Otherwise, to the best of our knowledge, there are no previous studies investigating ricotta cheese intake and LDL-C blood levels. Composition in terms of micro- and macronutrients might account for the results previously described, since Lorenzen and Astrup hypothesized a calcium-induced mechanism of increased fecal fat and bile acid elimination [30] that might influence cholesterol levels by decreasing cholesterol absorption. However, this mechanism could not fully explain the different influences of milk and ricotta cheese, since the latter has a significantly higher content of calcium in comparison to milk [31,32], so minerals and macronutrients might also influence serum blood lipids. First, it is important to note that ricotta has a greater phosphorus content than milk [31,32], and these higher levels might decrease the effect of calcium on cholesterol absorption through the calcium binding effect exerted by phosphorus itself. Even the sodium and potassium contents, lower and higher in milk, respectively [31,32], could have “a walk on part”, if any. Indeed, it was previously suggested that a lower sodium/potassium dietary intake ratio was correlated with decreased LDL-C blood levels [33]. Moreover, milk has a significantly lower content of lipids than ricotta, particularly saturated fatty acids (SFAs) [31,32], since it was observed that the influence of dietary SFAs on serum LDL-C was greater than that of cholesterol itself [34,35]. Furthermore, milk is richer in monounsaturated fatty acids than ricotta [31,32], and it was clearly demonstrated that unsaturated fatty acids, when used in substitution of SFAs, reduced LDL-C concentrations [36,37]. Intriguingly, although ricotta has a greater protein content than milk, its percentage of essential amino acids is lower in comparison to milk [31,32], as it was speculated that the essential amino acid intake could contribute to reducing LDL-C [38,39]. It is conceivable that all the proposed mechanisms, if taken alone, could not be sufficient to account for the different data; however, if the sum is greater than the parts, the whole nutrient composition might, at least in part, explain why milk and ricotta cheese impinge on LDL-C levels so differently. Altogether, although many studies claimed that dairy products were not correlated with LDL-C concentrations, it is not possible to draw definitive conclusions concerning this relationship. One may conceive that several confounding factors, different criteria for cohort recruitment and the relatively short period of intervention trials could account for these discrepancies. Hence, more studies, particularly long-lasting longitudinal and robust intervention studies, are warranted to fully elucidate this relationship.

Additionally, we reported that LDL-C serum concentrations were not affected by dietary factors, including the intake of fats (as a percentage of total energy intake) or that of cholesterol in the diet. Many studies have investigated whether cholesterol intake influences serum LDL-C, but the data are still controversial. In addition, a possible confounding factor might be that studies on this topic have a different prevalence of hyper- and hypo responders to dietary cholesterol. Two studies concerning a cohort of adult Chinese individuals reported that cholesterol intake accounted for increased LDL-C concentrations [40,41]; however, neither study adjusted their models according to SFAs. According to two recent meta-analyzes, LDL-C serum concentrations were positively correlated with habitual cholesterol intake [42] and increasing the amount of cholesterol consumption was positively associated with an increase in circulating LDL-C levels [43]. Conversely, in agreement with our results, Lin and colleagues observed no association between LDL-C blood levels and the habitual intake of dietary cholesterol [44], even after adjusting for SFAs. Interestingly, a recent survey carried out in a population cohort of nearly 40,000 Koreans reported that dietary cholesterol was significantly correlated with circulating LDL-C, but remarkably, this correlation disappeared after adjusting for SFA intake [45]. A possible explanation for the lack of influence of dietary factors in our study might be that the cohort we investigated was living in a Mediterranean area with dietary habits, as suggested by the FFQ, that can be considered healthy. Overall, it seems impossible to clearly establish this relationship due to a number of confounding factors that might alter the final data. In fact, other macronutrients, such as SFAs, fiber or calcium, can significantly influence LDL-C blood levels. This seems to be clearly demonstrated in the case of SFAs, which have been proven as pivotal determinants of serum LDL-C [34,35]. Moreover, previous data are also indicative of the presence of a significant threshold of cholesterol intake impinging on the development of LDL-C hypercholesterolemia [46].

In agreement with previous observations [47,48,49], our study demonstrates that LDL-C blood levels are independently associated with BMI, confirming that this is a possible contributing factor to the increased CV risk associated with obesity as suggested by the significantly higher values of c-IMT in the group with high LDL-C.

Furthermore, we found that level of HPA was not correlated with serum LDL-C. Our results are consistent with data from Huffman and coll. who reported that sedentary overweight individuals undergoing aerobic exercise did not show reduced LDL-C concentrations at the end of intervention [50], albeit they observed an improvement in LDL-C particle number and size [50]. On the contrary, Stefanick et al. reported that physical exercise was correlated with lower LDL-C blood levels [51] and overall, there is compelling evidence that regular physical exercise reduces circulating LDL-C [6]. However, according to data reported till now exercise only slightly affects LDL-C blood levels [6] and this small effect could account, at least in part, for the lack of correlation in our study. Nonetheless, the general recommendation for dyslipidemia management is to increase physical exercise [6].

This study has some strengths. The cohort recruited had characteristics similar, if not overlapping, to those of the general population. Additionally, we assessed habitual dietary patterns through a validated FFQ for the local population so that accuracy was granted as much as possible in investigating dairy food consumption. Moreover, our results should be scarcely influenced by confounding factors, since people with cardiovascular diseases or type 2 diabetes that may induce individuals to modify their dietary habits were excluded.

Our study also has important limitations. Due to the cross-sectional design of the study, we could not demonstrate any cause–effect relationship. Moreover, despite the resemblance between the characteristics of our cohort and those of the general population, the modality of cohort enrollment did not allow us to conclude that our cohort was fully representative of the general population. Indeed, we did not consider genetic factors that could influence LDL-C levels [52]. Finally, we did not adjust for the energy intake data concerning the consumption of the different foods. However, the FFQ returns approximate values of habitual food intakes that in some instances must be considered proxy indicators of different aspects of habitual diet. The accuracy of this tool is not adequate to calculate/adjust exactly the habitual amount of foods/energy.

## 5. Conclusions

In this study, we did not find any correlation between habitual dairy food consumption and serum LDL-C. However, we also reported that LDL-C blood levels were inversely and independently associated with cow milk intake, whereas the opposite occurred with ricotta cheese consumption. We hypothesize that different micro- and macronutrients in dairy products might account for this difference, especially SFA content. However, more studies, particularly robust RCTs assessing every aspect of dietary intake, are warranted to fully elucidate the relationship between LDL-C blood levels and dairy foods. Given the significant influence of body size on LDL-C concentrations, it is important that future studies focus on maintaining unaltered body parameters while evaluating the effect of dairy foods on circulating LDL-C.

## Figures and Tables

**Table 1:** Physical and clinical characteristics and dietary habits of the cohort according to the LDL-cholesterol blood levels.

	LDL-Cholesterol (mg/dL)		
	<130 *n* = 453	≥130 *n* = 349	*p* *
Age (years)	46 ± 15	55 ± 11	<0.001
Male sex (%)	36.0	39.5	0.30
Current smokers (%)	14.6	16.8	0.24
Body weight (kg)	74.0 ± 17.1	75.3 ± 15.1	0.28
BMI (kg/m^2^)	27.6 ± 5.9	28.5 ± 4.9	<0.05
Fat mass (%)	28.5 ± 9.8	30.0 ± 8.7	<0.05
Waist circumference (cm)	93.7 ± 14.2	97.3 ± 12.4	<0.001
WHR	0.89 ± 0.08	0.92 ± 0.08	<0.001
c-IMT (mm)	0.55 ± 0.17	0.64 ± 0.18	<0.001
Blood pressure (mmHg)			
systolic	119 ± 18	125 ± 16	<0.001
diastolic	78 ± 10	81 ± 10	<0.001
Level of HPA:			
Low (very low + low)	55.1	57.4	0.14
Habitual intake of:			
Lipids (% of EI)	30.5 ± 6.4	30.5 ± 6.4	0.97
Cholesterol (mg/day)	217 ± 89	206 ± 73	0.06
Milk (n. portions/week)	3.4 ± 3.9	2.9 ± 3.2	<0.05
Cheese (*n*. portions/week)	4.2 ± 2.3	4.3 ± 2.4	0.83
Ricotta cheese (*n*. portions/week)	0.7 ± 0.6	0.8 ± 0.7	<0.05
Total dairy products (*n*. portions/week)	9.6 ± 5.1	9.3 ± 5.0	0.41
Eggs (*n*./week)	1.2 ± 0.9	1.2 ± 0.9	0.93
Blood concentrations of:			
Glucose (mg/dL)	87.5 ± 16.5	88.6 ± 10.0	0.21
HbA1c (%)	5.3 ± 0.5	5.4 ± 0.4	<0.001
Insulin (µUI/mL)	9.4 ± 5.7	9.8 ± 5.9	0.27
Cholesterol (mg/dL)	182 ± 26	241 ± 30	<0.001
HDL-C (mg/dL)	62.6 ± 17.0	60.7 ± 16.1	0.11
Cholesterol/HDL-C	3.1 ± 0.8	4.2 ± 1.0	<0.001
Triglycerides (mg/dL)	85 ± 54	108 ± 51	<0.001
LDL-C (mg/dL)	102 ± 20	160 ± 24	<0.001
Uric acid (mg/dL)	4.7 ± 1.2	5.0 ± 1.3	<0.001
HOMA-IR	2.10 ± 1.40	2.21 ± 1.37	0.28

Mean ± SD; * Student’s unpaired *t*-test or χ^2^ when appropriate. BMI: body mass index; c-IMT: carotid intima-media thickness; EI: energy intake; HbA1c, glycated hemoglobin; HDL-C: high-density lipoprotein cholesterol; HOMA-IR: homeostatic model assessment of β-cell function and insulin resistance; HPA: habitual physical activity; LDL-C: low-density lipoprotein cholesterol; WHR: waist-to-hip ratio.

**Table 2:** Simple correlations among LDL-C blood concentrations, anthropometric variables and the habitual intake of nutrients and dairy foods.

	LDL-C	Age	Log BMI	Log Waist	Log Hip	WHR	FM %
LDL-C	-	0.33	0.14	0.19	0.06	0.23	0.10
		(<0.001)	(<0.001)	(<0.001)	(0.11)	(<0.001)	(<0.05)
Log Fat intake (% of EI)	0.02	0.02	0.02	−0.01	0.04	−0.07	−0.02
	(0.57)	(0.58)	(0.63)	(0.67)	(0.24)	(0.07)	(0.69)
Log Cholesterol intake (mg/day)	−0.07	−0.09	−0.04	−0.03	−0.02	−0.01	−0.03
	(0.07)	(<0.05)	(0.24)	(0.44)	(0.63)	(0.67)	(0.44)
Log Milk (portions/week)	−0.10	0.01	−0.01	−0.03	0.002	−0.05	0.01
	(<0.01)	(0.85)	(0.69)	(0.37)	(0.97)	(0.16)	(0.79)
Log Ricotta cheese (portions/week)	0.12	0.03	−0.04	−0.01	−0.06	0.04	−0.07
	(<0.001)	(0.44)	(0.26)	(0.75)	(0.11)	(0.25)	(0.09)
Log Dairy products (portions/week)	−0.02	0.04	0.04	0.06	0.06	0.03	0.03
	(0.56)	(0.29)	(0.24)	(0.12)	(0.10)	(0.48)	(0.47)

Pearson correlation coefficients (r), *p* values in parenthesis. BMI, body mass index; EI, energy intake; FM, fat mass; LDL-C, low-density lipoprotein cholesterol; WHR, waist-to-hip ratio.

**Table 3:** Multivariate stepwise (backward selection) regression analysis of factors correlated with blood LDL-cholesterol concentrations according to the data presented in Table 2.

	Coefficients	Standard Error	*p*
Age (years)	0.796	0.094	<0.001
Log BMI (kg/m^2^)	27.792	1.511	<0.001
Log Habitual intake of ricotta cheese (portions/week)	8.538	2.491	<0.001
Log Habitual intake of milk (portions/week)	−4.022	1.316	<0.005

R^2^ = 0.94. Variables not in the model: sex, waist circumference, WHR, FM%. BMI, body mass index; FM, fat mass; WHR, waist-to-hip ratio.

## Data Availability

Data available on request due to privacy restrictions. The data presented in this study are available on motivated request from the corresponding author.
